# Salvage treatment with plerixafor in poor mobilizing allogeneic stem cell donors: results of a prospective phase II-trial

**DOI:** 10.1038/s41409-020-01053-4

**Published:** 2020-10-07

**Authors:** Kristina Hölig, Helmuth Schmidt, Gero Hütter, Michael Kramer, Raphael Teipel, Katharina Heidrich, Kristin Zimmer, Falk Heidenreich, Matthias Blechschmidt, Tigran Torosian, Rainer Ordemann, Frank Kroschinsky, Elke Rücker-Braun, Laszlo Gopsca, Eva Maria Wagner-Drouet, Uta Oelschlaegel, Alexander H. Schmidt, Martin Bornhäuser, Gerhard Ehninger, Johannes Schetelig

**Affiliations:** 1 Department of Internal Medicine I, University Hospital Carl Gustav Carus, TU Dresden, Germany; 2 Cellex Collection Center GmbH, Cologne, Germany; 3 Cellex Collection Center GmbH, Dresden, Germany; 4 DKMS gemeinnützige GmbH, Clinical Trials Unit, Dresden, Germany; 5 Fundacja DKMS Polska, Warszawa, Poland; 6 MLL Münchner Leukämielabor GmbH, München, Germany; 7 National Institute of Hematology and Infectious Diseases, Department of Hematology and Stem Cell Transplantation, Budapest, Hungary; 8 Medizinische Klinik und Poliklinik III, Hämatologie, Internistische Onkologie, Pneumologie, Universitätsmedizin Mainz, Mainz, Germany; 9 DKMS gemeinnützige GmbH, Tübingen, Germany; 10 Center for Regenerative Therapies, Dresden, Germany

**Keywords:** Haematopoietic stem cells, Phase II trials

## Abstract

We conducted a prospective clinical trial to investigate the safety and efficacy of plerixafor (P) in allogeneic peripheral blood stem cells (PBSC) donors with poor mobilization response to standard-dose granulocyte colony-stimulating factor (G-CSF), defined by <2 × 10^6^ CD34 + cells/kg recipient body-weight (CD34+/kg RBW) after 1st apheresis. A single dose of 240 µg/kg P was injected subcutaneously at 10 p.m. on the day of the 1st apheresis. Thirty-seven allogeneic PBSC donors underwent study treatment. The median CD34+ count in peripheral blood was 15/µl on Day 1 after G-CSF alone, versus 44/µl on Day 2 after G-CSF plus P (*p* < 0.001). The median yield of CD34+ cells was 1.1 × 10^8^ on Day 1 and 2.8 × 10^8^ on Day 2. In contrast to a median yield of only 1.31 × 10^6^ CD CD34+/kg RBW on Day 1, triggering study inclusion, a median of 3.74 × 10^6^ CD CD34+/kg RBW were collected with G-CSF plus P on Day 2. Of 37 donors, 21 reached the target cell count of >4.5 × 10^6^ CD34+/kg RBW (57%, 95%CI 40–73%). No donor experienced a severe adverse event requiring treatment. In conclusion, P might be considered on a case-by-case basis for healthy allogeneic donors with very poor stem cell mobilization success after G-CSF.

## Introduction

More than 30,000 allogeneic hematopoietic cell transplantations are carried out worldwide annually [1]. In the last two decades, mobilization of peripheral blood stem cells (PBSC) has become the most common way to procure grafts for allogeneic transplantation from healthy related and unrelated donors. A sufficient cell dose represents a significant predictive factor for transplant outcomes [2]. A dose of ≥4.5 × 10^6^ CD34+ cells/kg recipient body-weight (RBW) is considered to be the optimal dose in the HLA-compatible transplant setting [3]. Cell doses between 2 and 4.5 × 10^6^ CD34 + cells/kg RBW have been demonstrated to be sufficient for hematologic recovery; however, patient survival was inferior compared to patients who had received higher cell doses [2, 4]. Conventional recombinant human granulocyte colony-stimulating factor (G-CSF)-based mobilization regimens result in unsatisfactory yields of CD 34+ cells from 2–5% of healthy allogeneic donors [5, 6]. Since alternative schedules for PBSC mobilization have not yet been approved, bone marrow collection remains the only salvage option. This approach presents additional risks for donors and increases the risk of insufficient bone marrow harvest. Plerixafor (Mozobil^®^ Genzyme Ltd., Haverhill, Suffolk, UK), a CXCR4-antagonist, was approved in 2008 for mobilization of PBSC in combination with G-CSF for patients with lymphoma or multiple myeloma [7]. Recently, the use of plerixafor for allogeneic PBSC mobilization in healthy allogeneic stem cell donors has been reported [8–12].

We conducted a prospective Phase 2 trial (MOBIL1) to systematically investigate the safety and efficacy of a single dose of plerixafor in healthy related and unrelated PBSC donors, who failed to donate ≥2 × 10^6^ CD34+ cells /kg RBW in the 1st apheresis after the routine administration of G-CSF.

## Donors and methods

### Study design

This multi-center Phase II study was conducted as an open label, uncontrolled, single arm trial with two stages. The study protocol was approved by the responsible Ethics Committees and regulatory authorities. The study was registered with ClinicalTrials.gov (NCT01954914).

### Routine mobilization and collection program

G-CSF mobilization and peripheral blood stem cell collection is generally conducted as outpatient procedure. Two doses of 7–10 µg/kg BW per day G-CSF (Lenograstim) are routinely administered subcutaneously to all PBSC donors. On the 5th day of G-CSF-mobilization, PBSC are collected via bilateral (anterior cubital and forearm) peripheral venous access by a continuous-flow blood cell separator. Four blood cell separators were used at the study sites: Cobe Spectra (Terumo BCT), Spectra Optia (Terumo BCT), COM.TEC (Fresenius Kabi), Amicus (Fresenius Kabi).

### Pre-screening

Donors were informed of the increased risk of poor mobilization at their first interview if they fulfilled two or more of the following criteria: Female donors aged over 40 years; donor body-weight (BW) < 70 kg; ratio of RBW to donor BW > 130%; donor platelet count <200 × 10^9^/l ^6^. For these planned donations, the corresponding transplant centers were informed in advance of the possibility of an insufficient cell dose and asked if they would accept a graft mobilized with G-CSF and plerixafor.

### Study enrollment

Three collection sites participated in the study. Donors, who donated less than 2.0 × 10^6^ cells/kg RBW at the 1st apheresis after 5 days of G-CSF-administration, could be enrolled. Key eligibility criteria were: age between 18 and 75 years; medical clearance in place for allogeneic PBSC donation; no thoracic discomfort or symptomatic splenomegaly; platelet count ≥80 × 10^9^/L, serum creatinine < 80 μmol/l for female donors or <106 μmol/l for male donors; no contraindication against a second leukapheresis. Written informed consent of the donors was obtained after the result of the 1st apheresis was available and written approval of the transplant center was obtained to accept plerixafor-mobilized stem cells from the second apheresis.

### Study treatment

**Fig. 1:**
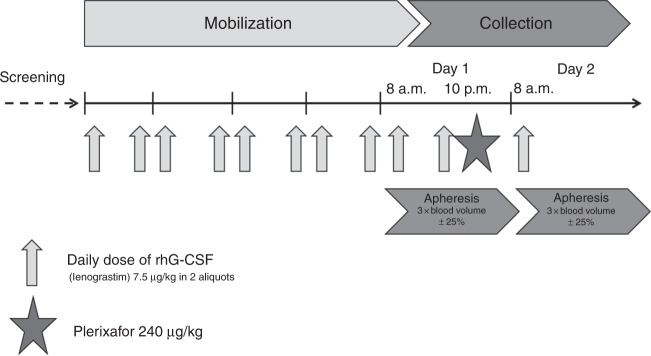
Study scheme.

An overview of the study design is provided in Fig. 1. Study participants donors returned to the clinic in the evening of the day of the 1st apheresis (Day 1) and received a single subcutaneous injection of plerixafor at a dose of 240 μg/kg of donor BW at 10 p.m. by a trained nurse. The donors were monitored for 30 min after the injection. Vital parameters and potential side effects were documented. Thereafter donors spent the night outside the clinic. Administration of G-CSF was continued according to the routine schedule. The 2nd leukapheresis was performed at 8 a.m. on the next day (Day 2). The same separator was used for both collections. During each leukapheresis, three times the donor’s blood volume (±25%) had to be processed within 4 h. Heparin (2.500–5.000IE/donor) and ACDA (ratio 1:19) were used as anticoagulants.

### Laboratory tests

Complete Blood Counts (CBC) and CD34+ counts in the peripheral blood of the donors were measured prior to each apheresis session. The CD 34+ counts in the peripheral blood and the leukapheresis products were analyzed by a “single platform“ technique (according to Sutherland et al.) locally and at the central lab at the University Hospital Dresden [13].

### Follow-up

Before administration of the study drug, after the 2nd apheresis and 30 days after PBSC collection, the donor was interviewed and completed a questionnaire. Adverse events were graded according to the National Cancer Institute Version 3.0 Common Terminology Criteria for Adverse Events (Cancer Therapy Evaluation Program, Common Terminology Criteria for Adverse Events, Version 3.0, DCTD, NCI, NIH, DHHS March 31, 2003 (http://ctep.cancer.gov), Publication Date: August 9, 2006). Long-term follow-up with annual standardized telephone interviews was scheduled for a period of five years from donation.

### Data collection and statistics

The primary endpoint was the rate of successful PBSC donations, defined by two products collected during the 1st and 2nd apheresis which together contained more than 4.5 × 10^6^ CD34+ cells/kg RBW. Any other outcome was defined as failure. We assumed a 20% improvement over an arbitrarily chosen success rate of 50%. The sample size was calculated for a two-stage Minimax Design according to Simon, maintaining a one-sided type 1 error of 5% and a power of 80% [14]. In total, it was necessary to recruit 23 donors in Stage 1 and 14 donors in Stage 2. The secondary objectives included the analysis of the safety of plerixafor administration, the success of mobilization and the composition of the apheresis products from Day 1 and Day 2. For continuous variables compatibility with normal distribution and variance homogeneity were checked using graphical methods. Median and interquartile range are presented for continuous variables. Peripheral CD34 + counts from Day 1 and Day 2 were compared with a paired *t*-test. Comparisons of leukocyte counts and lymphocyte counts between independent groups were done with the Wilcoxon Rank test, as their distribution was not compatible with the normal distribution. Further on, we fitted multivariable linear regression models to identify prognostic factors for the relative increase of CD34+ cell counts and the total yield of CD34+ cells per kg RBW after administration of plerixafor. Age, sex, donor weight, smoking status, and counts for leukocytes, lymphocytes, monocytes, CD34 + cells and platelets were entered into the model. The ratio of donor to patient weight was tested for influence on the total CD34+ yield per kg RBW. Survival probabilities were estimated with the Kaplan–Meier method. To compare survival with published data, the published survival probabilities were weighted with the observed probabilities of the disease risk categories in our study. The confirmatory primary efficacy test was one-sided. Exploratory tests were two-sided with 5% confidence level. No adjustment for multiple testing was done for exploratory tests.

## Results

### Donor characteristics

Between 1/2014 and 12/2015, a total of 39 healthy allogeneic unrelated PBSC donors were screened, and signed informed consent forms for the MOBIL1 trial. No related donor was enrolled into the study. Two donors did not receive study medication: One donor did not meet the inclusion criterion for the CD34+ yield at the 1st apheresis and it was necessary to exclude the second donor due to fever. In total, 37 donors received study medication. During the study period, PBSC collections from 6293 allogeneic donors were performed at the three participating centers, hence the study collective represented 0.6% of the whole donor population. The pre-screening algorithm indicated more donors to be at risk for insufficient mobilization, but the yield of the 1st apheresis eventually was equal or above 2.0 × 10^6^ CD 34+ cells/kg RBW and thus the donors did not meet this eligibility criterion [6]. Donor and recipient characteristics are shown in Table 1. Notably, median recipient weight equaled 123% of the median donor weight.

**Table 1:** Donor and recipient characteristics.

Donor characteristics	Total number, *N* = 37
Gender (male/female)	17 (46%)/20 (54%)
Age (years), median (IQR)	34 (26–48)
Height in cm, median (IQR)	176 (167–182)
Weight in kg, median (IQR)	69 (61–76)
Smoker	15 (41%)
Recipient characteristics	
Weight in kg, median (IQR)	85 (74–102)

*IQR* interquartile range.

### Mobilization efficacy and graft quality

**Fig. 2:**
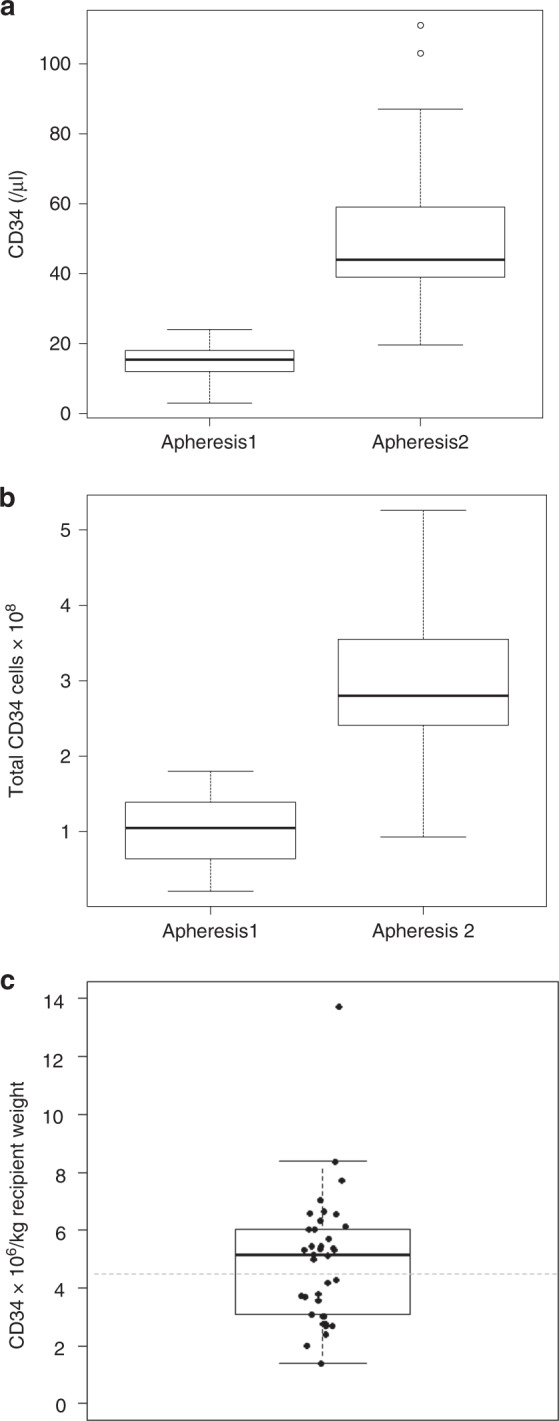
CD34+ cell counts in the peripheral blood and the leukapheresis products before and after mobilization with Plerixafor. The box plots represent the CD34+ cell counts in the peripheral blood of the donor (**a**) and in the collected leukapheresis product (**b**) on Day 1 and Day 2, respectively. **c** shows the total CD34+ yields collected on Day 1 and Day 2. Each dot represents the result of one donor. Altogether 15 donations failed to reach the target cell count of >4.5 × 10^6^ CD 34+ cells per kg recipient body weight (represented by the dotted gray line). The median count is represented by a bold line, the boxes represent the interquartile range and the whiskers represent 1.5 times the interquartile range. Outliers are plotted as dots.

The median CD 34+ counts in peripheral blood on Day 1 before the 1st apheresis after G-CSF alone were 15/µl (IQR 12–18). Following the administration of G-CSF and plerixafor, the median peripheral CD 34+ count was 44/µl (IQR 38–61) on Day 2 (paired *t*-test, *p* < 0.001; Fig. 2a). This is equal to a 2.9-fold increase in the CD 34+ count. All aphereses were conducted via bilateral peripheral venous access. The parameters of both leukaphereses and the collected products are presented in Table 2. Technical parameters (processed blood volume, duration of apheresis, and volume of the product) were comparable between the 1st and 2nd leukapheresis.

**Table 2:** Comparison of apheresis procedures and cell products.

Variable	1^st^ apheresis median (IQR)	2^nd^ apheresis median (IQR)	Ratio of medians (2^nd^ /1^st^)	Wilcoxon-signed-rank-test for paired samples (*p* value)
Blood volume processed [L]	12.8 (11.4–14.4)	13.0 (11.7–14.4)	1.0	0.455
Blood volume processed [× TBV]	2.98 (2.48–3.07)	2.85 (2.46–3.21)	0.96	0.455
Separation time [min]	193 (172–218)	195 (178–223)	1.0	0.874
TNC [×10^8^]	475 (405–642)	854 (687–1109)	1.8	<0.001
Volume of product [ml]	257 (195–382)	295 (234–433)	1.1	0.003
TNC [10^6^ per ml]	187 (160–223)	289 (248–348)	1.5	<0.001
MNC [× 10^8^]	419 (319–490)	622 (552–754)	1.5	<0.001
Hematocrit [%]	3 (2–5)	3 (2–6)	1.0	0.014
Vitality CD34 cells [%]	100 (99–100)	100 (100–100)	1.0	0.392
CD 34+ [×10^8^]	1.05 (0.64–1.41)	2.80 (2.32–3.71)	2.7	<0.001
CD 34+ [×10^6^/kg^a^]	1.31 (0.8–1.65)	3.74 (2.26–4.69)	2.9	<0.001
Total amount of CD34+ cells [×10^6^/kg RBW]	5.16 (3.06–6.10)		-	-
CD 3 [%]	31 (27–40)	31 (23–35)	1.0	<0.001
CD3 cells [×10^8^]	162 (113–197)	246 (197–295)	1.5	<0.001

*TBV* total body blood volume, *TNC* total nucleated cells, *MNC* mono-nuclear cells, *IQR* interquartile range, *RBW* recipient body weight.

^a^Body weight of the recipient body weight.

A higher number of Total Nucleated Cells (TNC) and Mono-Nuclear Cells (MNC) could be collected during the 2nd leukapheresis compared to the 1st leukapheresis. Consequently, the median total CD 34+ counts in the apheresis products were significantly higher: 1.1 × 10^8^ (IQR 0.6–1.4) on Day 1 and 2.8 × 10^8^ (IQR 2.3–3.7) on Day 2 (Table 2 and Fig. 2b), equivalent to a 2.7-fold increase in yield.

Altogether, a median number of 5.2 × 10^6^ (IQR 3.1–6.1) CD 34+ cells per kg RBW were collected in both aphereses. Twenty-one out of 37 donors (57%, 95% confidence interval 40–73%) reached the target CD34 + cell count of a CD34 + cell dose of >4.5 × 10^6^ CD 34+ cells per kg recipient BW. However, the null hypothesis of a greater than 50% success rate could not be formally rejected at the 5% level. The total collection yields of all donors are shown in Fig. 2c.

While the primary endpoint referred to a target CD34+ count of 4.5 × 10^6^ per kg RBW, many centers consider 4 × 10^6^ per kg RBW adequate. With this target number 23/37 aphereses had been considered successful (62%, 95% confidence Interval 45–78%).

The median cell dose in terms of the donor weight instead of the recipient weight was 5.6 × 10^6^ (IQR 4.3–7.6) CD34+/kg. When using donor weight as a reference, 27 donors (73%) reached the target cell count of >4.5 × 10^6^/kg.

Notably, the five donors with the poorest mobilization result on G-CSF alone had donated ≤1.0 × 10^6^ CD 34+ cells per kg RBW (range, 0.2–1.0) during the 1st apheresis. Their CD 34+ cell counts in the peripheral blood ranged between 5 and 8 CD34+ cells/µl prior to the first apheresis. After administration of plerixafor, the CD 34+ cell counts increased to between 40–62/µl, approximating to an eightfold increase, and the median CD 34+ cell yield in the 2nd leukapheresis product was 12-times higher than for the 1st product. Remarkably, only one of these five donors finally had a total yield of less than 4.5 × 10^6^ CD 34+ cells per kg RBW after the second leukapheresis (range, 2.4–13.7 × 10^6^ CD 34+ cells per kg RBW). No donor underwent bone marrow collection and there was no 2nd donation request because of graft failure in a recipient.

### Prognostic factors for mobilization success with plerixafor

Given the extraordinarily good mobilization success of plerixafor in those donors with the poorest mobilization after G-CSF alone, we performed a risk factor analysis. In univariate comparisons, donors with a higher relative increase in CD34+ cells had lower leukocyte counts and lower lymphocyte counts prior to administration of plerixafor (Wilcoxon Rank test, *p* = 0.02 and *p* = 0.01, respectively). In the multivariable linear regression model, the CD34+ cell count in the peripheral blood on Day 1 (positively correlated) and the leukocyte count (negatively correlated) had a significant impact on the log-fold change of the peripheral CD34+ cell count, and the interaction between these two factors was found to be significant. We found the same pattern of results when we fitted regression models for the CD34+ count per kg RBW of the 2nd leukapheresis as the dependent variable.

In order to describe the interaction of these two risk factors, we grouped donors by their baseline counts prior to plerixafor using the median CD34 + cell count (15.4/µl) in the peripheral blood and the median WBC count (27.6 × 10^9^/L) as thresholds into CD34+*-high/-low* and WBC *-high/-low* donors, and compared their mobilization success. The WBC*-low* and CD34*+ low* donors (*N* = 14) had the biggest mobilization success (fivefold increase of CD34+ cells in the blood and sixfold increase of the CD34+-yield). All remaining donors had substantially smaller mobilization effects (an approximate threefold increase of CD34+ cell counts in the blood and of the CD34+ cell-yield).

Remarkably, donor age did not significantly affect mobilization response after the administration of plerixafor. Yet, the mobilization success in female donors was slightly better (3.6-fold increase of CD34+ cells in the blood) than in male donors (2.7-fold increase).

### Cell composition

The TNC and MNC counts of the 2nd leukapheresis product after mobilization with G-CSF plus plerixafor were 1.5-fold higher (854 versus 475 × 10^8^ TNC and 622 versus 419 × 10^8^ MNC, *p* < 0.001 respectively). The percentages of CD3+ cells were identical in both products. Due to the higher TNC count of the 2nd leukapheresis, the absolute CD3+ count was higher in the 2nd leukapheresis product. This increase in total numbers was observed for most of the analyzed lymphocyte populations. The total number of T-cells was found to be increased in the 2nd leukapheresis product, with CD8+ T-cells showing a higher gain (1.7-fold) compared to a 1.4-fold increase of CD4+ T-cells. A higher increase (1.9-fold) was found in B-cell counts. Notably, the absolute number of Natural Killer cells did not differ between the two products.

### Safety

One aspect of donor safety during PBSC collection is constituted by the changes in blood counts between various time points of the procedure (before and after administration of plerixafor and after the second leukapheresis). These parameters are displayed in Table 3, together with the values after the 30-day follow-up.

**Table 3:** Blood counts prior to and after the 2nd apheresis procedure.

Parameter (IQR)	1^st^ leukapheresis	2^nd^ leukapheresis	Follow-up 30d
WBC count before LPH [×10^9^/L]	27.6 (23.1–37.5)	50.5 (38.9–56.5)	4.2 (3.5–4.9)
WBC count after LPH [×10^9^/L]	27.4 (24.3–34.5)	47.0 (36.0–53.3)	
Hb before LPH [g/dl]	13.5 (12.7–14.2)	13.0 (12.3–14.0)	13.0 (12.3–14.0)
Hb after LPH [g/dl]	12.9 (12.1–13.9)	13.5 (12.6–14.7)	
PLT count before LPH [×10^9^/L]	185 (172–220)	119 (98–135)	197 (183–240)
PLT count after LPH [×10^9^/L]	116 (94–128)	75 (65–89)	

*IQR* interquartile range, *d* days, *WBC* white blood cell count, *L, LPH* leukapheresis, *Hb* hemoglobin, *PLT* platelet.

Administration of plerixafor nearly doubled the WBC compared to the value after G-CSF alone. Platelet count after 5 days of G-CSF was already within the lower normal range ahead of the 1st leukapheresis (185 × 10^9^/L; IQR 172–220). After the second leukapheresis, the median platelet count was still 75 × 10^9^/L (IQR 65–89). The follow-up evaluation 30 days after PBSC collection revealed values in the normal range.

**Fig. 3:**
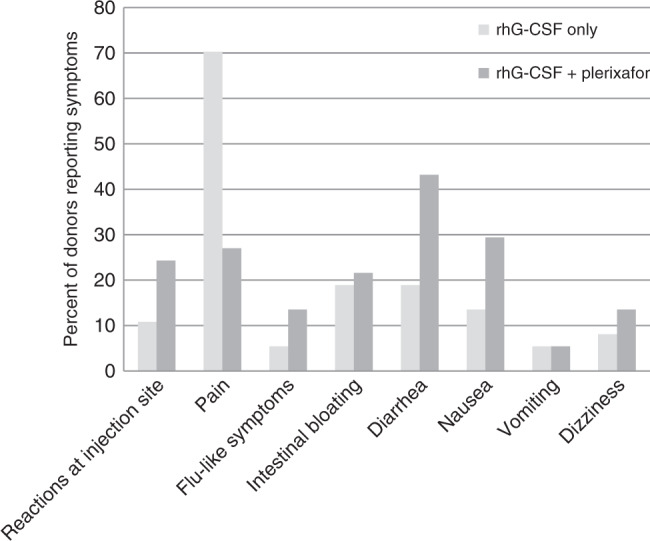
Adverse event rates reported for mobilization with rhG-CSF only versus rhG-CSF plus Plerixafor. The histogram shows percentages of donors with selected adverse events during stem mobilization with G-CSF (light grey bars) versus G-CSF plus plerixafor (dark grey bars). Side effects of mobilization with G-CSF and G-CSF + Plerixafor.

**Fig. 4:**
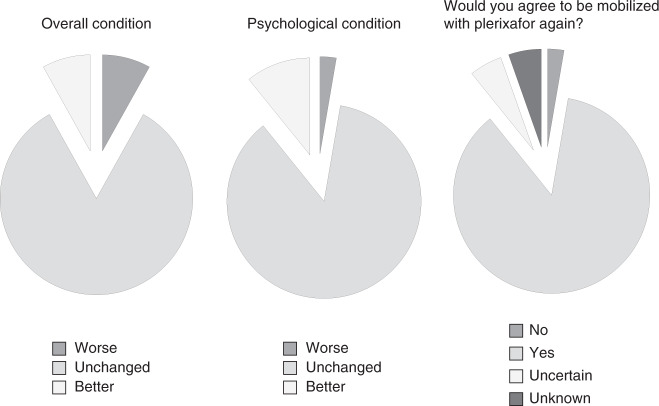
Day 30 self-assessment of the mobilization procedure by the donors. The pie charts represent self-assessments of the overall and psychological condition on Day 30 after cell collection and an evaluation of the investigational stem cell mobilization from the donor perspective. Self Assessment of Mobilization Procedure.

Adverse Events (AE) were classified according to the CTCAE V3.0 Criteria, and are listed in Table 4. The majority of AE which occurred were mild or moderate (Grade 1 or 2). In two donors (5%) Grade 3 thrombocytopenia was diagnosed after the 2nd apheresis. No donor experienced signs of bleeding as a consequence of thrombocytopenia. The comparison between the side effects of the mobilization with G-CSF alone and the combination of G-CSF and plerixafor is illustrated in Fig. 3. The main side effects of G-CSF were bone pain and headache (summarized as pain). The additional application of plerixafor changed the spectrum of side effects towards gastrointestinal symptoms, like nausea and diarrhea. General tolerability of the study treatment was also expressed in terms of general donor well-being 30 days after donation. The results of the interviews are shown in Fig. 4. Only 8% of donors reported a worse overall condition compared to the state before the mobilization. Thirty-two donors (86%) would agree to receive plerixafor again. Only one donor would refuse a second donation after mobilization with G-CSF and plerixafor. This donor had experienced a Grade 3 thrombocytopenia after the 2nd apheresis and had suffered from a Grade 1 citrate reaction and Grade 1 diarrhea. Four donors were irresolute with respect to a second donation.

**Table 4:** Adverse events during the study period.

Adverse event	Total number *n* (%)	CTCAE Grade 1 *n* (%)	CTCAE Grade 2 *n* (%)	CTCAE Grade 3 *n* (%)
Citrate reaction	23 (62)	18 (49)	5 (14)	
Diarrhea	15 (41)	10 (27)	5 (14)	
Pain	14 (38)	9 (24)	5 (14)	
Nausea	12 (32)	8 (22)	4 (11)	
Intestinal bloating	9 (24)	6 (16)	3 (8)	
Dizziness	7 (19)	2 (5)	5 (14)	
Flu-like symptoms	5 (14)	2 (5)	3 (8)	
Thrombocytopenia	4 (11)		2 (5)	2 (5)
Vomiting	4 (11)	4 (11)		
Itching at injection site	2 (5)	2 (5)		
Heat sensation	1 (3)	1 (3)		
Erythrocyturia^a^	1 (3)		1 (3)	
Pneumonia	1 (3)	1 (3)		
Elevated PTT	1 (3)	1 (3)		
Insomnia	1 (3)	1 (3)		
Swelling at injection site	1 (3)	1 (3)		
Tachycardia	1 (3)	1 (3)		

*CTCAE* Common Toxicity Criteria for Adverse Events, *n* number, *PTT* partial thromboplastin time.

^a^Grade 2 erythrocyturia occurred in a 29-year old donor with a platelet count of 135 × 10^9^/L after the 2^nd^ leukapheresis and resolved spontaneously.

### Patient outcomes

Outcome data are available for 25 patients (68%) who received the PBSC which were donated within the MOBIL1 trial. Engraftment data of 18 patients have been reported before [15].

The median patient age was 56 years (range, 10–74 years). The Karnofsky Performance status prior to transplantation was good in 83% of patients and restricted in 17% of patients. Disease risk, classified according to the Disease Risk Index (DRI) [16], was high or very high for 44% of patients. Seventeen donors (68%) were matched for HLA-A, -B, -C, and -DRB1 but eight donor-recipient pairs (32%) were mismatched. All 25 patients engrafted. The median time to neutrophil engraftment >0.5 × 10^9^/L was 18 days (interquartile range, 16 to 20 days) and to platelet engraftment >20 × 10^9^/L was 17 days (interquartile range, 13–30 days). The Day +100 cumulative incidence of acute GVHD grades II–IV and III–IV were 50% (95%-CI, 34–75%) and 25% (95%-CI, 13–50%). By degree of HLA-compatibility, the Day +100 incidences of acute GVHD grades III–IV were 18% (95%-CI, 6–49%) among patients with well matched HLA-compatible donors and 43% (95%-CI, 18 to 100%) among patients with partially matched unrelated donors. The Day +28 mortality and Day +100 mortality were 0% (95%-CI, 0–14%) and 8% (95%-CI 1–26%), respectively. The survival probability at 2 years after transplantation was 66% (95%-CI, 49–89%). This compares to a predicted 2-year overall survival of 44% for a virtual population of patients sharing the same patients’ characteristics as the CIBMTR Disease Risk Index study, and a weighted disease risk according to the patient population here [16].

## Discussion

The CD34+ cell count of the graft may influence outcome after allogeneic PBSC transplantation. Independent of the goal to reach optimal PBSC doses for the recipient, donor safety remains of utmost importance. For this reason, procedures with a higher potential risk or inconvenience for the donors should be avoided. This reasoning applies to multiple large volume aphereses with the risk of thrombocytopenia, as well as bone marrow collection immediately after ineffective PBSC donation.

Plerixafor offers a promising opportunity to increase PBSC mobilization associated with little physical stress for the donors. Initial clinical trials with plerixafor as a single mobilization agent in healthy volunteers and related allogeneic PBSC donors have shown promising results [8, 17]. Unfortunately, the capacity of plerixafor as a single agent is lower than the standard G-CSF-regimen and ~30% of healthy donors do not mobilize an acceptable number of CD34+ cells [8, 17]. In an attempt to overcome this problem, the route of administration was changed from subcutaneous injection to intravenous infusion and the dose of plerixafor was doubled [11, 18]. Both strategies did not show a striking success even in healthy donors who were randomly assigned. Yet, combinations of plerixafor and G-CSF might give a good chance to reach the target PBSC yield in poorly mobilizing donors. Only case reports and small case series have been reported in the current literature, where plerixafor was given as a salvage strategy to poorly mobilizing healthy allogeneic donors who failed to mobilize sufficiently with single-agent G-CSF [10, 19].

Here, we present data of a prospective trial where plerixafor was evaluated as salvage treatment for poorly mobilizing healthy PBSC donors. Due to the stringent definition of poor mobilization (<2 × 10^6^ CD34 + cells/kg RBW in the 1st apheresis) the study collective represented a highly selected population of stem cell donors accounting for only 0.6% of all stem cell donors. This group of donors presented a challenge in medical care and counseling. Overall, 57% (95%-CI, 40–73%) of these donors finally managed to donate >4.5 × 10^6^ CD34 + cells/kg RBW. After a single dose of plerixafor, the median CD34+ count in peripheral blood increased 2.9-fold and the CD34+ cell yield increased 2.7-fold. While the rate of successful mobilization with plerixafor was somewhat smaller than expected, our data still demonstrated the feasibility of salvage treatment with plerixafor added to G-CSF in allogeneic poor mobilizing donors.

Importantly, the increase of mobilization efficacy was even higher in those donors who represented the biggest challenge. Donors who had CD34 + cell counts of less than 15.4/µl and leukocyte counts of less than 27.6 × 10^9^/L after mobilization with G-CSF had a fivefold increase of CD34 + cells in the peripheral blood, and a sixfold increase of the CD34+-yield. While the possibility of poor compliance with G-CSF administration in “WBC*-low* and CD34 + *low”* donors cannot be ruled out, poor mobilization due to pharmacogenetic variation of the complex process of G-CSF mobilization is another explanation. Polymorphisms in stromal cell-derived factor 1 (SDF-1), CD44, and the relaxin receptor have all been implicated with mobilization efficacy [20–24]. Although there is a lack of large confirmatory studies to confirm these findings, pharmacogenetic variation of G-CSF activity is likely. Thus, addition of a drug with a different mechanism of action for poor mobilizing donors is well founded.

The MOBIL1 study was designed in such a way that the clinical need was addressed. For that reason the target yield was defined by a cell dose with respect to recipient weight, not donor weight. The median weight of the patients was 85 kg (IQR, 74–102 kg) and the median weight of the donors was only 69 kg (IQR 61–76 kg). This difference reflects the challenge for the donors.

It cannot be expected that ad hoc salvage treatment with plerixafor after failed G-CSF mobilization will be successful in every poor mobilizing donor [25]. Earlier addition of plerixafor (e.g., starting on Day 3 of G-CSF application) might be more efficacious in some donors. Similar to data in the autologous setting, earlier application of plerixafor would possibly increase the chance of optimal mobilization while shortening G-CSF exposure time for donors. In addition, new drugs and next generation CXCR4 inhibitors should be tested with respect to their potential for PBSC mobilization [26, 27]. However, any pre-emptive approach depends on a reliable prediction of poor mobilization with G-CSF alone.

Considering demographic data and CBC parameters, our algorithm did not reveal a reliable tool to identify poor mobilizers before G-CSF application [6]. Besides genetic polymorphisms, other parameters (e.g., basal CD34+ count) might also be useful predictors of mobilization performance [28–31].

To compare the results we analyzed donors who donated at the three trial sites prior to the start of the MOBIL1-trial as historical controls (see Table 5). The G-CSF dosage was identical. The median age of poor mobilizing donors (<2 × 10^6^ CD 34+ cells/kg RBW in 1st apheresis) was 38 years (IQR, 28 to 46 years) compared to 36 years (IQR 27–42 years) of good mobilizing donors (*p* = 0.046). Body weight and gender correlated strongly with mobilization efficacy (*p* < 0.001). The mobilization data of 168 poor mobilizing donors are shown in Table 5. With continued mobilization with G-CSF alone the number of CD 34+ cells collected in the 2nd apheresis was nearly always lower than the yield of the first stem cell collection. Administration of plerixafor clearly changed this pattern. With plerixafor CD 34+ yields of the 2nd apheresis were remarkably better than of the 1st collection (3.74 versus 1.15 × 10^6^ CD 34+ cells/kg RBW). This resulted in much higher success rates of donors who received plerixafor compared to G-CSF alone (57% in this trial versus 7% in controls).

**Table 5:** Comparison of CD 34 yields of the study population and historical controls.

Variable	1^st^ apheresis median (IQR) MOBIL-1	2^nd^ apheresis median (IQR) MOBIL-1	1^st^ apheresis median (IQR) historical controls	2^nd^ apheresis median (IQR) historical controls
CD34 + [×10^8^]	1.05 (0.64–1.41)	2.80 (2.32–3.71)	1.27 (1–1.5)	0.9 (0.65–1.29)
CD34 + [×10^6^/kg RBW]	1.31 (0.8–1.65)	3.74 (2.26–4.69)	1.63 (1.32–1.84)	1.15 (0.8–1.57)
Total amount of CD34 + cells [x10^6^/kg RBW]	5.16 (3.06–6.10)		2.77 (2.28–3.27)	
Donors reaching target >4.5 ×10^6^/kg	21/37 (57%, 95%CI 40–73%)		11/168 (7%, 95%CI 6–16%)	
Donors reaching target >4.0 × 10^6^/kg	23/37 (62%, 95%CI 45–78%)		18/168 (11%, 95%CI 3–11%)	

Data on 168 healthy allogeneic donors who donated <2 × 10^6^ /kg in 1^st^ apheresis at the trial sites before 2012.

*IQR* inter quartile range, *RBW* recipient body weight, *CI* confidence interval.

Administration of plerixafor was well tolerated. Only two CTCAE Grade 3 adverse events (thrombocytopenia after 2nd apheresis in both cases) occurred throughout the trial. The transient decrease of platelet counts must be interpreted as a consequence of the apheresis procedure itself. In the MOBIL1 trial, we limited the processed blood volume to improve donor safety and to allow for two aphereses with comparable parameters. Our previous approach was a large volume apheresis on day 5 and a limited apheresis volume on day 6 in donors with low platelet counts. With this procedure platelet counts in poor mobilizing donors were already very low after the 1st apheresis and a 2nd collection was not permitted. In future, poor mobilizing donors could be identified by measuring the CD34 count on day 4 to allow preemptive administration of plerixafor on the evening before the 1st apheresis.

All remaining adverse events were mild or moderate. Some complaints occurred as frequently on Day 1 as on Day 2 and even symptoms which are typically associated with plerixafor, such as vomiting and intestinal bloating, occurred with comparable frequencies on both days. Local reactions, nausea and diarrhea, however were reported more often after plerixafor, whereas pain was more pronounced after G-CSF alone. A good indicator of the tolerability of a new treatment is the subjective assessment of the affected individuals. The overall condition 30 days after donation was unchanged or better in 92% of donors and 86% would agree to receive the study drug again. These data are aligned with our experience with donor follow-up after mobilization with G-CSF alone [32].

When considering the risk-benefit ratio of a single subcutaneous injection of plerixafor compared to a salvage bone marrow harvest in general anesthesia, CXCR4 inhibition appears to be advantageous. In a historical donor population, the incidence of urgent bone marrow harvest was about 0.2% of the donors in the participating centers. Other researchers and clinicians also considered the use of plerixafor as salvage treatment for allogeneic stem cell donation and published case reports and case series [10, 19, 33]. In line with our data, no serious adverse events were documented in these case reports.

From an economic perspective, the costs of urgent bone marrow harvest at our hospital currently are ~70% of the cost of the approach using plerixafor. This price might differ substantially in other countries.

When considering the poor patient-characteristics of the graft recipients with respect to their DRI, performance status and the high rate of partially matched donors, overall outcomes after transplantation compare favorably. The median time to ANC recovery of 18 days is relatively long for PBSC grafts. However, but the sample size is small and the patients were treated heterogeneously, so that no conclusions can be drawn from this observation. The key message is that no graft failure had been reported. Acute GVHD grades III-IV was experienced by 25% of patients in this study, however. In recent publications on patients who had received plerixafor-mobilized allogeneic PBSC from HLA-identical siblings, the rate of acute GVHD grade III-IV was also significant, ranging between 12 and 17% [11, 12]. When the different graft composition with respect to immune effector cell populations is considered [15, 34, 35], controlled trials are especially warranted to formally establish non-inferiority with respect to endpoints such as acute GVHD potentially arising from plerixafor plus G-CSF mobilized PBSCT, compared to G-CSF mobilized PBSCT. This is particularly important when the different graft composition with respect to immune effector cell populations is considered [15, 34, 35].

Taken together, plerixafor appears to be a promising salvage option for poorly mobilizing allogeneic donors with a good risk-benefit ratio [36]. Although not approved for this indication in the US and Europe, administration of plerixafor might be considered on a case-by-case basis in healthy allogeneic donors with very poor stem cell mobilization success after G-CSF. At the time of writing, the World Marrow Donor Association has not yet accepted this option.

Besides the optimization of classical G-CSF-based regimens, other concepts for stem cell mobilization are in clinical and preclinical development [37]. Particularly CXCR2 agonists [38] and VLA4 antagonists [39] are interesting candidates for short-time mobilization after single injections. The combination of these substances resulted in a rapid mobilization within 15–30 min after injection in mice [39]. These new agents could allow augmenting or even replacing standard mobilization strategies and open novel perspectives to overcome poor mobilization. Thus, further research in this area is highly warranted in the best interest of donors and recipients.
