# Correction to: Salvage treatment with plerixafor in poor mobilizing allogeneic stem cell donors: results of a prospective phase II-trial

**DOI:** 10.1038/s41409-021-01523-3

**Published:** 2021-11-12

**Authors:** Kristina Hölig, Helmuth Schmidt, Gero Hütter, Michael Kramer, Raphael Teipel, Katharina Heidrich, Kristin Zimmer, Falk Heidenreich, Matthias Blechschmidt, Tigran Torosian, Rainer Ordemann, Frank Kroschinsky, Elke Rücker-Braun, Laszlo Gopsca, Eva Maria Wagner-Drouet, Uta Oelschlaegel, Alexander H. Schmidt, Martin Bornhäuser, Gerhard Ehninger, Johannes Schetelig

**Affiliations:** 1 Department of Internal Medicine I, University Hospital Carl Gustav Carus, TU Dresden, Germany; 2 Cellex Collection Center GmbH, Cologne, Germany; 3 Cellex Collection Center GmbH, Dresden, Germany; 4 DKMS gemeinnützige GmbH, Clinical Trials Unit, Dresden, Germany; 5 Fundacja DKMS Polska, Warszawa, Poland; 6 MLL Münchner Leukämielabor GmbH, München, Germany; 7 National Institute of Hematology and Infectious Diseases, Department of Hematology and Stem Cell Transplantation, Budapest, Hungary; 8 Medizinische Klinik und Poliklinik III, Hämatologie, Internistische Onkologie, Pneumologie, Universitätsmedizin Mainz, Mainz, Germany; 9 DKMS gemeinnützige GmbH, Tübingen, Germany; 10 Center for Regenerative Therapies, Dresden, Germany

**Keywords:** Haematopoietic stem cells, Phase II trials

Correction to: *Bone Marrow Transplantation* 10.1038/s41409-020-01053-4

The Salvage treatment with plerixafor in poor mobilizing allogeneic stem cell donors: results of a prospective phase II-trial, written by Kristina Hölig, Helmuth Schmidt, Gero Hütter, Michael Kramer, Raphael Teipel, Katharina Heidrich, Kristin Zimmer, Falk Heidenreich, Matthias Blechschmidt, Tigran Torosian, Rainer Ordemann, Frank Kroschinsky, Elke Rücker-Braun, Laszlo Gopsca, Eva Maria Wagner-Drouet, Uta Oelschlaegel, Alexander H. Schmidt, Martin Bornhäuser, Gerhard Ehninger, Johannes Schetelig was originally published Online First without Open Access. After publication in volume 56, issue 3, page 635–645 the author decided to opt for Open Choice and to make the article an Open Access publication. Therefore, the copyright of the article has been changed to © The Author(s) 2020 and the article is forthwith distributed under the terms of the Creative Commons Attribution 4.0 International License, which permits use, sharing, adaptation, distribution and reproduction in any medium or format, as long as you give appropriate credit to the original author(s) and the source, provide a link to the Creative Commons licence, and indicate if changes were made. The images or other third party material in this article are included in the article’s Creative Commons licence, unless indicated otherwise in a credit line to the material. If material is not included in the article’s Creative Commons licence and your intended use is not permitted by statutory regulation or exceeds the permitted use, you will need to obtain permission directly from the copyright holder. To view a copy of this licence, visit http://creativecommons.org/licenses/by/4.0. Open access funding enabled and organized by Projekt DEAL.

